# Formation of Thick Immersion Coatings and Residual Stress Evaluation in the System ZrB_2_–ZrO_2_: Experimental and Numerical Investigation

**DOI:** 10.3390/ma16020781

**Published:** 2023-01-12

**Authors:** Ales Buyakov, Igor Smolin, Valentina Zimina, Nikita Fedyanin, Vasiliy Shmakov, Svetlana Buyakova

**Affiliations:** 1 Institute of Strength Physics and Materials Science SB RAS, 634055 Tomsk, Russia; 2 School of Advanced Manufacturing Technologies, National Research Tomsk Polytechnic University, 634050 Tomsk, Russia

**Keywords:** functional ceramics, reliability, immersion coating, cold plasma treatment, microstructural study, thermal stress, graded composite, FEM

## Abstract

The combination of various oxide ceramics in layered and functionally graded composites allows for the development of novel materials, including for high-temperature applications. This study demonstrates the possibility of obtaining a thick ZrO_2_-based coating on a ZrB_2_–SiC ceramic substrate by the immersion method. For better wettability, the porous ZrB_2_–SiC substrate is treated with cold plasma without changing the structure and phase composition of the surface. Immersion of the substrate in a ZrO_2_-based slurry results in the formation of a gradient transition layer due to ZrO_2_ particle penetration into the pore volume. The interfacial residual microstresses are evaluated experimentally. The residual macrostresses in the samples are calculated by finite element simulation. It is shown that the thermal residual stresses in the ZrB_2_–SiC substrate are compressive and do not exceed 43 MPa. In the ZrO_2_ coating and transition layers of the composite, the residual stresses are tensile. Their values increase as they get closer to the outer layer of the ZrO_2_ coating and reach 1525 MPa. This confirms the conclusions about the presence of tensile residual stresses made in the experimental part of the work when observing crack propagation in the surface layers during indentation.

## 1. Introduction

Ceramic coatings, including on ceramic substrates, are conventionally applied using PVD and CVD methods. The advantage of these technologies is that the thickness of the applied coating can be controlled with high accuracy. For some applications, however, this is a drawback because the thickness of such coatings can hardly reach several microns, while its increase can lead to the loss of adhesion between layers, delamination, cracking due to thermal residual stresses, and higher deposition costs [1,2].

A less trivial task is the formation of transition and gradient coatings with high adhesion strength and high spalling resistance. These issues were discussed in studies on ultra-high-temperature ceramics (UHTC), where laminated composites consisting of components with different thermal expansion coefficients were obtained by pressure co-sintering of layers with different phase ratios, or by plasma spraying [3,4]. Such composites are important for the thermal protection of high-energy facilities, and further research in this area is aimed at reducing the thermal conductivity of the composite and increasing its fracture toughness due to the formation of numerous interfaces.

In recent years, the research focus in the field of UHTC has shifted from the characteristics and parameters of carbon/carbon composites and composites based on monolithic carbides and borides with uniformly distributed functional particles towards approaches of the modern structural design of composite materials. Some authors have shown that the targeted design of architecture significantly improves the performance of materials, including their ability to adequately respond to external loads. An example of such materials is layered composite ceramics with higher oxidation and ablation resistance, whose surface layers prevent oxygen diffusion to less oxidation-resistant inner layers due to the formation of refractory oxides, oxynitrides, oxycarbides, and other compounds [5,6,7,8].

Along with the high melting point, the efficiency of thermal protection materials is determined by the thermal conductivity. Biamino et al. [9] showed that the introduction of highly porous layers into a sandwich-structured composite leads to a two-fold decrease in its bulk thermal conductivity, which ranges from 0.53 to 0.27 cm^2^/s at room temperature and from 0.15 to 0.08 cm^2^/s at 900 °C. Zirconium and hafnium oxides are known to have extremely low thermal conductivity, but these materials are not suitable because of their polymorphism and phase transformations at high temperatures. Tetragonal stabilized zirconia is the most promising due to the high impact strength achieved by transformation hardening. At temperatures above 2000 °C (up to 2500 °C, depending on the stabilizer content), tetragonal ZrO_2_ evolves to cubic with a smaller unit cell volume, giving rise to second-order stresses, structural defects, and a significant loss of strength.

The above limitation can be overcome by using fully stabilized cubic ZrO_2_, which is stable up to the melting point. Long-term operation of such thermal protection materials at extreme temperatures inevitably results in the migration of elements that create oxygen vacancies in the ZrO_2_ structure towards grain boundaries, as well as destabilization as the temperature decreases further. That is why the vast majority of extreme operating conditions do not imply the long-term use of thermal protection materials based on borides and carbides of group IV and V metals.

As shown earlier, the production of layered and graded functional materials is technologically difficult. UHTC composites with uniform particle distribution in the matrix are successfully obtained by slip, tape, or gel casting [10], including additive manufacturing via direct ink writing [11], while the fabrication of layered and graded high-temperature composites with complex structure and geometry is still a matter of discussion.

Bao et al. [12] proposed an immersion method to form a thick Al_2_O_3_-based surface layer on ZrO_2_ ceramics. A pre-sintered (1200 °C) porous ZrO_2_ sample was immersed in an alumina slurry composed of Al_2_O_3_ powders, water, dispersant, and defoamer, after which it was sintered at 1500 °C. A microstructural study showed no delamination or discontinuities in the obtained coating, whose thickness amounted to at least 50 μm, and the residual microstresses generated at the ZrO_2_–Al_2_O_3_ interface due to different thermal expansion coefficients increased the damage tolerance of the material.

Conventionally, the immersion method is used to form thick polymer coatings, including on metal substrates. For example, Prosolov et al. [13] successfully applied a calcium phosphate coating to Ti samples. However, this approach is used much less frequently for ceramic materials, which may be due to their low wettability. Nonetheless, it has been demonstrated that cold plasma surface modification of ceramics can improve wettability [14].

Plasma treatment technology is widely used for surface modification of various materials. High-temperature plasma treatment can modify the structure of the substrate surface, e.g., during surface modification of polymers and metals [15,16]. In the case of low-temperature plasma treatment (below 773 K) of ionic and ionic-covalent ceramics, the changes are obviously caused only by the short-term effect of ion precipitation from the gaseous medium and a local increase in surface energy.

It is known that residual stresses arising in layered and graded materials (in particular, between the coating and the substrate) due to a mismatch in the thermal expansion coefficient between layers are one of the most important factors affecting the mechanical properties and durability. For example, compressive residual stresses in tensile materials can increase their resistance to cracking and delamination. Therefore, the determination of residual stresses and understanding their formation mechanisms are necessary for the quality control of products made from such materials. Parameters such as temperature, layer thickness, and porosity can be used to control residual stresses in materials and, consequently, to adjust their behavior during operation. At present, the residual stress measurements are the subject of extensive research. Various residual stress measurement methods have been proposed for experimental studies, including optical, X-ray, and other approaches [17,18]. Theoretical studies employ various numerical models [19,20,21] and analytical models [22,23,24,25].

This work presents a case study to validate the possibility of applying thick immersion ZrO_2_-based coatings to ZrB_2_ ceramics and to evaluate the interfacial microstresses. Control over the zone of different-sign elastic stresses at the coating–substrate interface can help to modify the impact strength and cracking behavior of the composite. The advantage of such composites for practical applications is the abnormally low thermal conductivity of ZrO_2_, which is capable of acting as, e.g., the back layer of a ZrB_2_-based heat shield. The difficulty of the problem being solved lies in the different thermal expansion coefficients of these compounds, which inhibits their co-sintering and leads to low wettability, which prevents the build-up of immersion coating.

## 2. Materials and Methods

### 2.1. Materials

The investigation was performed on disk-shaped ceramic composite samples with a thickness of 5 mm and a diameter of 30 mm. The samples were fabricated from commercially available powders of ZrB_2_ with an average particle size of 15.7 ± 11.2 μm and SiC, used as a sintering additive, with an average particle size of 3.1 ± 1.9 μm. Powder mixtures of ZrB_2_–30% SiC were mixed and activated in a planetary ball mill with ZrO_2_ grinding media. The pre-sintered ceramic samples were compacted by cold uniaxial pressing of ZrB_2_–30% SiC powder mixture (hereinafter ZrB_2_–SiC) and 1 wt % polyvinyl alcohol solution, followed by sintering in a vacuum furnace at 1600 °C. The residual porosity of the pre-sintered samples was 48%.

The low wettability of ZrB_2_ was overcome by air cold plasma treatment of the sample surfaces. The cold plasma treatment of pre-sintered samples was carried out in a laboratory setup at a frequency of 1000 Hz and a pulse energy of 0.32 J in air while rotating a glass drum with the samples at a speed of 60 rpm.

**Figure 1:**
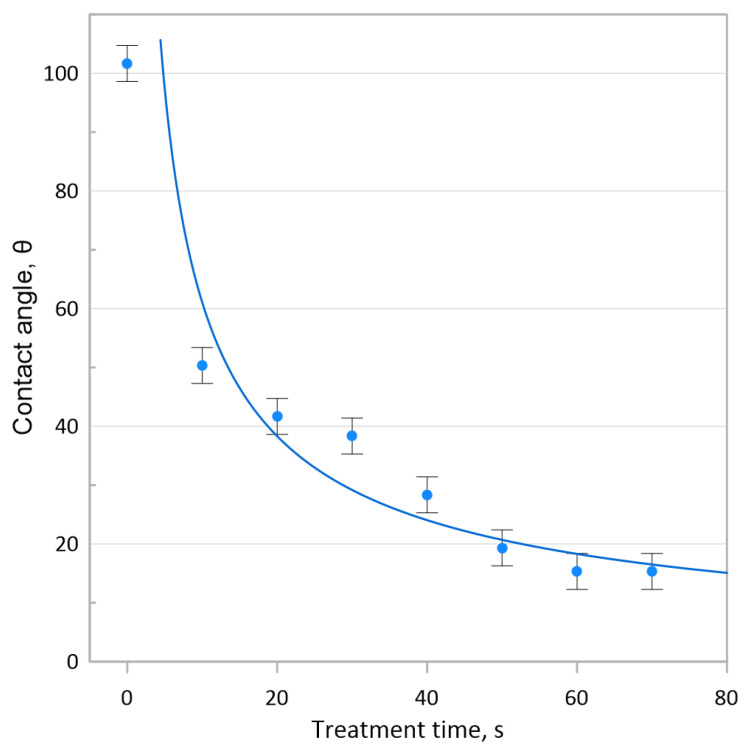
Dependence of the contact angle variation of a distilled water drop on the surface of the control ZrB_2_–SiC sample versus cold plasma treatment time.

The contact angle variation was evaluated using a control ZrB_2_–SiC sample with a high relative density of 99.97% (Figure 1). Contact angle measurements of a water drop on the surface of the control ZrB_2_ sample with high relative density showed a steady increase at treatment times up to 50 s (Figure 1). Further exposure to cold plasma did not lead to noticeable changes in surface wettability. After several hours of sample exposure in air outside the protective gas atmosphere, the observed increase in contact angle vanished. Therefore, immersion coatings were applied to samples of pre-sintered porous ZrB_2_–SiC ceramics exposed to cold plasma for 50 s.

**Figure 2:**
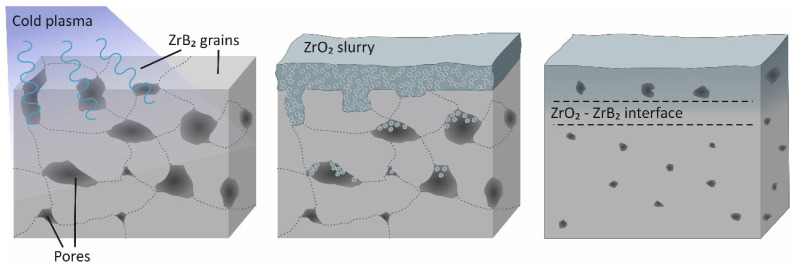
Schematic of the immersion ZrO_2_ coating formation on pre-sintered ZrB_2_–SiC.

Coatings on the pre-sintered ceramic samples were formed by immersion in a slurry composed of ZrO_2_ nanoparticles (10 g/30 mL), distilled water, and dispersant (0.2 g/30 mL) under sonication for 10 min (Figure 2). The ZrO_2_ powder used in the study was obtained by plasma chemical synthesis and stabilized with 3 mol % MgO. The morphology of nanostructured ZrO_2_ particles varied from hollow spheres and sphere fragments to agglomerates of size < 1 μm and spheroidized agglomerates of size up to 10 μm. The time between the end of cold plasma treatment of pre-sintered ZrB_2_–SiC samples and the complete immersion of the samples in the slurry was less than 10 s.

The samples coated with the ZrO_2_ layer were placed in a graphite crucible on a surface coated with inert boron nitride to prevent sticking of the test sample to the substrate, and were then transferred to a vacuum furnace. The time of transfer of the coated samples to the vacuum furnace for sintering was no more than 30 min. The ZrB_2_–SiC samples coated with ZrO_2_ were sintered in vacuum at a temperature of 1800 °C without isothermal holding.

### 2.2. Methods

#### 2.2.1. Experimental Methods

The ceramics microstructure was studied by scanning electron microscopy (Vega 3 SH, Tescan) with secondary and backscattered electron imaging. The porosity and pore size distribution were measured on the polished cross section of a test sample by applying the point-counting method as well as using the linear intercept method according to ASTM E112.

The phase composition was studied by X-ray diffraction analysis. XRD analysis was carried out on a DRON diffractometer (BOUREVESTNIK, JSC) in the angular range 20–80° with a step of 0.03° and an exposure of 3 s using CuKα filtered radiation. XRD patterns were recorded directly from the coating surface, which was polished with 10 to 0.5 μm diamond pastes to remove a 5-μm thick layer in order to improve the quality of the surface microstructure. Further X-ray diffraction analysis was performed after the removal of 25-µm thick surface layers. The obtained XRD patterns were analyzed using Match! Version 3.1 software (Crystal Impact) and cards 00-034-0423 (ZrB_2_) and 04-011-9021 (ZrO_2_) of the International Centre for Diffraction Data.

The magnitude of the second-order microstresses acting in the ceramics structure was determined from XRD peak broadening as the product of the microstrain value [26] and the Young’s modulus of the corresponding phase at room temperature.

The initiation of microcracks and the crack propagation behavior were studied by Vickers indentation along the ZrO_2_–ZrB_2_–SiC interface at a load of 100 N, Duramin-5 (Stuers A/S).

#### 2.2.2. Numerical Methods

In view of the cylindrical symmetry of the samples, the problem can be considered in a two-dimensional axisymmetric formulation. The *z* symmetry axis is directed perpendicular to the disk plane, and the *r* axis is directed along the disk radius. All stress-strain parameters along the third *θ* axis are assumed to be constant because the layers are homogeneous.

The system of equations in the adopted formulation includes equilibrium Equations (1), strain-displacement relations (2), constitutive equations (Duhamel–Neumann relations) (3), and heat conduction Equation (4) [27,28,29]:

(1) $$\frac{\partial \sigma_{rr}}{\partial r}+\frac{\partial \sigma_{zr}}{\partial z}+\frac{\sigma_{rr}-\sigma_{\theta \theta }}{r}=0,\;\frac{\partial \sigma_{rz}}{\partial r}+\frac{\partial \sigma_{zz}}{\partial z}+\frac{\sigma_{rz}}{r}=0,$$

(2) $$\varepsilon_{rr}=\frac{du_{r}}{dr},\;\varepsilon_{zz}=\frac{du_{z}}{dz},\;\varepsilon_{\theta \theta }=\frac{u_{r}}{r},\;\varepsilon_{rz}=\frac{1}{2}\left(\frac{du_{r}}{dz}+\frac{du_{z}}{dr}\right),$$

(3) $$\sigma_{ij}=2\mu \varepsilon_{ij}+\delta_{ij}\left[\lambda \varepsilon_{kk}-3K\alpha \left(T-T_{0}\right)\right],$$

(4) $$c_{\varepsilon }\rho \frac{\partial T}{\partial t}=\frac{1}{r}\frac{\partial }{\partial r}\left(r\lambda_{T}\frac{\partial T}{\partial r}\right)+\frac{\partial }{\partial z}\left(\lambda_{T}\frac{\partial T}{\partial z}\right).$$

Here $\sigma_{ij}$ is the stress tensor component, $\varepsilon_{ij}$ is the strain tensor component, $u_{r}$, $u_{z}$ are the displacement vector components, λ and μ are the Lame parameters, *K* is the bulk modulus, α is the linear thermal expansion coefficient, *T* is the current temperature, $T_{0}$ is the temperature of the initial state (sintering temperature), $c_{\varepsilon }$ is the heat capacity at constant strains, ρ is the density, and $\lambda_{T}$ is the thermal conductivity. Stresses, strains, and displacements are functions of spatial coordinates. Material parameters (Young’s modulus, Poisson’s ratio, and linear thermal expansion coefficient) are also functions of coordinates.

For the initial conditions, we assume that there are no initial stresses and strains. Due to symmetry, we consider ¼ of the disk cross section. The boundary conditions are set based on the conditions of axial symmetry at *r* = 0, plane symmetry at *z* = 2.5 mm, and free boundary conditions on the outer surfaces of the disk (*z* = 5 mm and *r* = 15 mm):

(5) $$\sigma_{ij}n_{j}=0.$$

For the heat conduction equation, Newton’s conditions for the heat flux were set on all outer surfaces:

(6) $$q=-\beta \left(T-T_{r}\right),$$

and zero heat flux was set on the axes of symmetry:

(7) q=0,

where β is the heat transfer coefficient, and $T_{r}$ is the room temperature.

At the interfaces between different layers, the condition of ideal mechanical and thermal contact was assumed to be fulfilled.

The numerical simulation of the stresses and strains during sample cooling was performed by the finite element method in a two-dimensional axisymmetric formulation, implemented in Abaqus/Standart software. The temperature dependences of elastic moduli and linear thermal expansion coefficients were taken into account.

**Figure 3:**
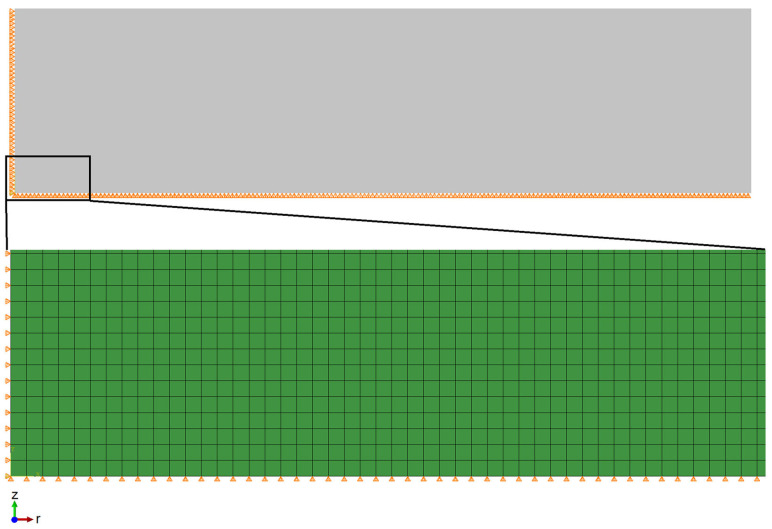
Geometry and mesh of the numerical model.

A finite element model was developed using a mesh with axisymmetric four-node coupled temperature-displacement elements (CAX4RT) as shown in Figure 3. To obtain favorable and precise simulation results, the mesh contained 1,000,000 elements.

## 3. Results and Discussion

### 3.1. Experiments

The plasma treatment method used in this work is characterized by a low temperature relative to the ZrB_2_ crystallization temperature, which prevents structural changes in the ceramic surface. It is known, however, that plasma treatment in an air atmosphere can lead to the precipitation of hydrophilic functional groups on the surface of ceramics [14] and therefore increase the wettability of the ZrB_2_ surface, including inside open subsurface pores. An attempt to deposit a ZrO_2_ coating on an untreated ZrB_2_–SiC substrate with reduced wettability led to the formation of cracks at the coating–substrate interface. The coating adhesion to the substrate turned out to be extremely weak or even absent in some regions, so that the coating detached from the sample after removal from the vacuum furnace. A similar result was reported in a study where a composite based on ceramics with different thermal expansion coefficients was fabricated by methods without pressure sintering, such as hot pressing or spark plasma sintering [30].

The microstructural study of the polished cross-sectional surface showed that the thickness of the formed ZrO_2_ layer was about 50 μm. The coating porosity was about 5%. The residual porosity of ZrB_2_–SiC after sintering was less than 10%. Pores in the ZrO_2_ layer were characterized by a much lower density but a larger size as compared to ZrB_2_. The nanostructured ZrO_2_ powder obtained by plasma chemical synthesis had a high packing density, which prevented cracking of the structure during shrinkage. However, the presence of voids in hollow spherical powder particles and between large spheroidized agglomerates resulted in the formation of micro- (<500 nm) and macropores (>10 µm) after sintering. Macropores could also be due to the high sintering temperature, relative to the homologous temperature of ZrO_2_, and a long time of thermal treatment sufficient for the micropores to coalesce [31].

**Figure 4:**
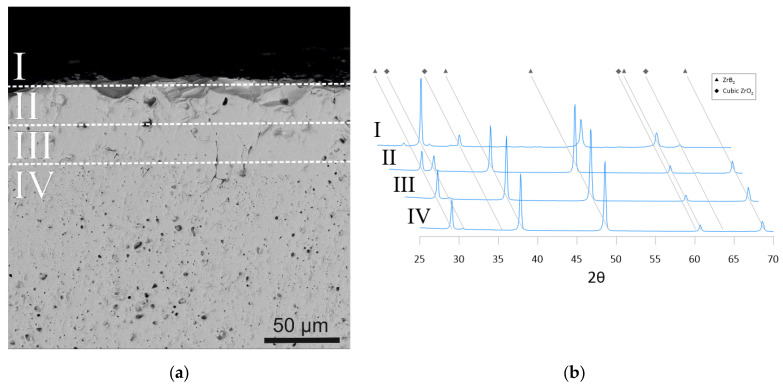
Change in the phase composition at the interface between the ZrO_2_ coating and ZrB_2_–SiC substrate: (**a**) SEM image of the studied ceramic sample with an indication of the zones where the surface XRD patterns were obtained; (**b**) XRD patterns of subsurface layers showing changes in the phase composition.

The phase composition of the coating surface is represented by cubic ZrO_2_ and low-intensity reflections of the monoclinic modification (Figure 4). At a distance of 25 μm from the surface, the phase composition consists mainly of ZrB_2_ and cubic ZrO_2_ (32%). At a distance of 50 and 75 µm from the surface, the ZrO_2_ reflections become barely distinguishable. The gradient phase composition in the interfacial region between the ZrO_2_ coating and ZrB_2_–SiC substrate was determined by open subsurface pores in the pre-sintered ZrB_2_–SiC sample, where the pores and pore channels were large enough for the ZrO_2_-based slurry to penetrate. Furthermore, the high diffusivity of oxygen and boron contributes to the redistribution of these elements in the contact region at high sintering temperatures, resulting in a strong mechanical bond, a smeared interface, and a small amount of interfacial defects [32,33].

**Figure 5:**
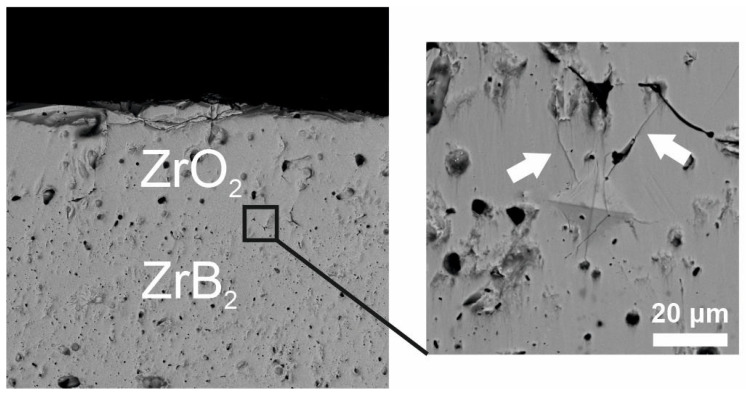
A Vickers indent on the cross-sectional surface at the interface between the ZrO_2_ coating and ZrB_2_–SiC substrate.

**Figure 6:**
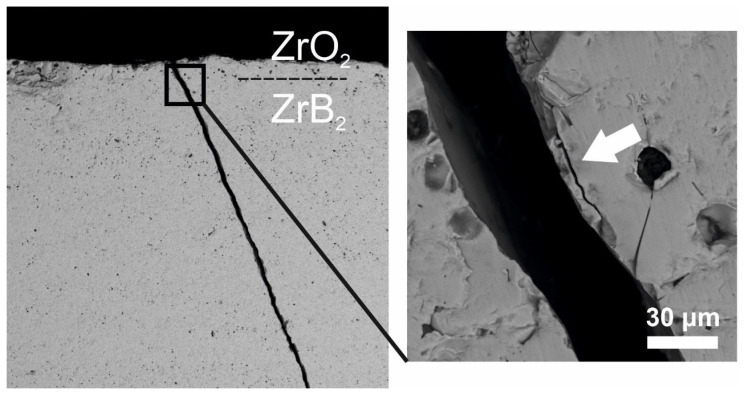
Crack propagation behavior in the ZrO_2_ layer on the ZrB_2_–SiC substrate.

Vickers indentation along the ZrO_2_–ZrB_2_–SiC interface led to the formation of microcracks in cubic ZrO_2_, which has lower fracture toughness than ZrB_2_, and no interfacial cracks were observed (Figure 5). A similar result was obtained in three-point bending of the graded ceramic composite: when a crack reached cubic zirconia, it bifurcated, but the formed microcracks were directed parallel to or slightly deviated from the applied load axis, and therefore the zirconia surface layer did not detach (Figure 6).

**Figure 7:**
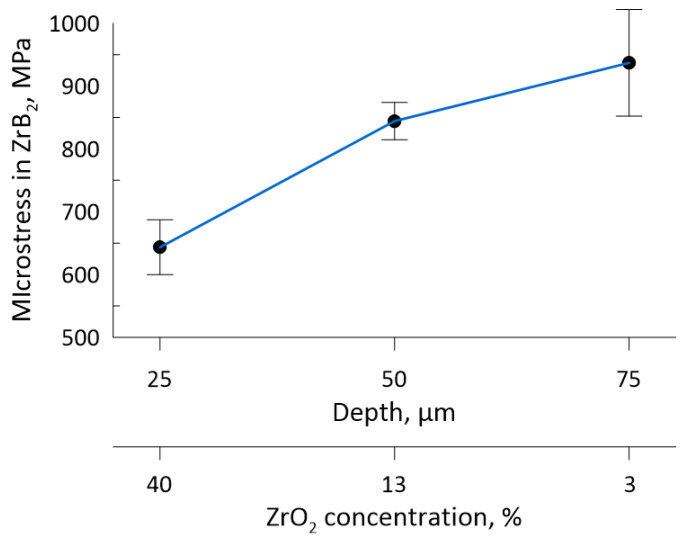
Variation of microstresses in ZrB_2_ with distance from the surface of the studied ceramic sample.

The result obtained can be explained by the generation of second-order elastic microstress fields in the ZrO_2_–ZrB_2_ gradient layer, which is due to the different thermal expansion coefficients of the components. Since the ZrO_2_ reflection intensity at high diffraction angles of the XRD pattern is insufficient, it is impossible to empirically estimate the stresses arising in the oxide. The dependence of the microstress values in ZrB_2_ on the distance from the surface of the test sample is shown in Figure 7. According to the numerical simulation shown below, the decrease in microstresses in ZrB_2_ with increasing ZrO_2_ content may be caused by a change in the sign of stresses.

The literature contains many studies on the generation and evaluation of residual stresses in composite ceramics due to different CTEs of the components. Some authors used the effect of the CTE difference to increase the impact strength of brittle ceramics. This effect was achieved by increasing the work of a propagating crack in the region of compressive stresses. In other cases, the difference in the CTEs between the matrix and the inclusion can lead to multiple microcracking but without sample failure [34,35,36]. At the same time, a multiple increase in the surface area also increases the required energy of the main macrocrack to propagate through the stressed region [37,38].

Presumably, the observed pattern of different-sign elastic stresses at the coating–substrate interface can have a favorable effect on the impact strength of the composite as a whole. Multiple bifurcation of a crack propagating in the coating should undoubtedly result in the partitioning and dissipation of its energy and, with a high probability, crack arrest.

### 3.2. Numerical Simulation

**Figure 8:**
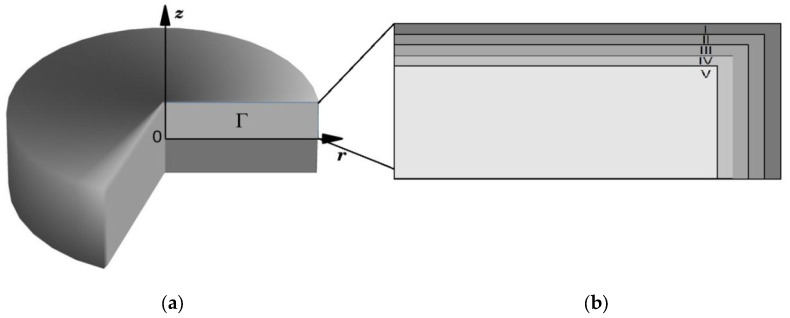
Schematic diagram of the ceramic composite: (**a**) Disk-shaped composite sample; (**b**) Modeling area.

The residual macrostresses arising in the studied samples were evaluated in a numerical study of a porous ZrB_2_–30 vol % SiC ceramic disk with a multilayer oxide coating with different ZrO_2_ contents, cooled from sintering temperature to room temperature. A schematic illustration of the composite sample structure is presented in Figure 8.

In accordance with the experimental data, we study a disk-shaped composite sample of thickness 5 mm and diameter 30 mm consisting of five layers of different composition. All the layers are assumed to be homogeneous. Due to symmetry, we consider ¼ of the disk cross section (region Γ in Figure 8a). The geometric model of the composite used in the numerical simulation is presented in Figure 8b. The thickness of the layers is specified from the experimental data: the thickness of layers I to IV is 0.025 mm, and that of layer V is 2.4 mm. The porosity of layers I to IV is taken to be 5%, and that of ZrB_2_–SiC layer V is 11%.

For residual stress analysis, we calculated the elastic moduli (Young’s modulus, Poisson’s ratio), thermal expansion coefficients, coefficients of thermal conductivity and heat capacity, and densities of the composite components, taking into account the temperature variation of these physical and mechanical properties. The elastic and thermophysical characteristics of ZrO_2_ and ZrB_2_–SiC were determined based on the temperature dependences taken from Refs. [39,40]. The effective properties of the intermediate layers of the composite were determined by the mixture rule.

It was also necessary to account for the effect of the porosity of the layers on the effective mechanical and thermal characteristics of the material. The effect of porosity on Young’s modulus value (*E^eff^*) was described using the exponential relationship proposed in Ref. [41]:

(8) $$E^{eff}=E^{M}\exp(-2\theta /(1-\theta )).$$

Here, *E^M^* is the Young’s modulus of the matrix (pore-free ceramics), and θ is the porosity.

The effective value of Poisson’s ratio of porous ceramics was determined using the following relationship [42,43]:

(9) $$\nu^{eff}=0.014+(1-\theta /0.472)(\nu^{M}-0.014),$$

where ν*^M^* is the Poisson’s ratio of the matrix.

Analysis of the literature [44,45,46] revealed that porosity does not affect the linear thermal expansion coefficient of most materials. Therefore, we assumed here that α^eff^ is independent of porosity in the gradient ceramic composite under study.

Table 1 gives the values of the physical and mechanical properties of the composite layers at the sintering temperature (*T*_0_ = 1800 °C) used in the calculations.

**Figure 9:**
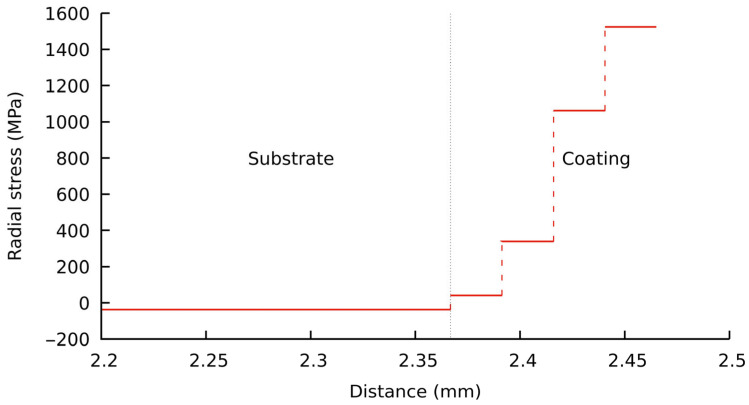
Through-the-thickness distribution of residual stresses in the central part of the disk.

**Figure 10:**
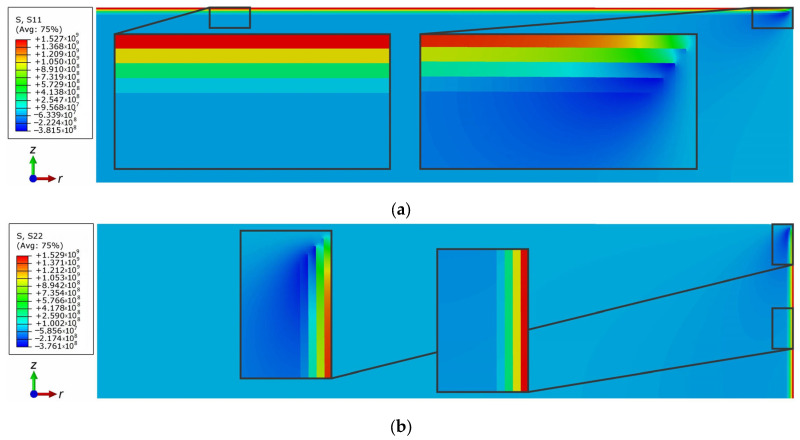
Distributions of the normal stress components: (**a**) Radial stress σ*_rr_*; (**b**) Axial stress σ*_zz_*.

Figure 9 and Figure 10 present the numerical simulation results for the stress state of the studied composite during cooling. The curve of radial stress distribution through the disk thickness in Figure 9 is discontinuous, where the discontinuities are indicated by dotted vertical lines. It can be seen that the thermal residual stresses in the ZrB_2_–SiC layer are compressive and their value does not exceed 43 MPa. In all other layers of the composite, the residual stresses are tensile. Their values increase as they get closer to the outer ZrO_2_ layer and reach 1525 MPa.

It should be noted that the stress distribution curve will not be stepwise, as in Figure 9, but smooth, in accordance with the given law of ZrO_2_ content variation, if the concentration of zirconium dioxide in the surface layer changes smoothly from a specified value (30%) on the surface to zero at a depth of approximately 100 mm.

Two-dimensional distributions of the normal stress components are shown in Figure 10. One can see that the residual stresses differ in different layers. They are both negative (compressive) and positive (tensile). The stresses in the disk layers (sections) remain unchanged up to a region at the upper edge of the disk.

The maximum positive stresses are observed in the upper layer of the modeled ZrO_2_ coating. As in the experiment, the residual stresses change sign from compressive stresses in the substrate (in the bulk of the ZrB_2_–SiC disk) to tensile stresses closer to the surface of the composite sample. This confirms the conclusions about the presence of tensile residual stresses in the coating, made in the experimental part of the work when observing the crack growth in the surface layers during indentation.

## 4. Conclusions

This proof-of-concept study demonstrated the possibility of applying thick coatings and fabricating graded ceramic/ceramic composites with significantly different thermal expansion coefficients of the substrate and coating materials. The proposed approach was validated on a composite sample consisting of a ZrB_2_–SiC substrate and ZrO_2_ coating. It was shown that a gradient coating on a porous substrate can be formed by immersion in a slurry of fine-grained powders and using cold plasma treatment that increases the wettability but does not change the structure and phase composition of the surface, which was confirmed by X-ray diffraction analysis.

The residual stresses in the graded ceramic/ceramic composite were evaluated numerically. Thermal residual stresses in the ZrB_2_–SiC substrate are compressive, whereas they are tensile in the ZrO_2_ coating and transition layers, according to simulations. This is corroborated by the experimental data, which showed that growing cracks bifurcate upon reaching the oxide layer of the composite.

## Figures and Tables

**Table 1:** Physical and mechanical properties of the composite layers.

Layers	Layer Composition	Young’s Modulus, GPa	Poisson’s Ratio	Coefficient of Linear Thermal Expansion α × 10^6^, K^−1^	Specific Heat, J/(kg∙K)	Thermal Conductivity, W/(m∙K)	Density, kg/m^3^
I	60%ZrO_2_ + 40%(ZrB_2_–SiC)	245.4	0.267	10.06	801.2	24.32	5020
II	40%ZrO_2_ + 60%(ZrB_2_–SiC)	270.0	0.227	9.305	809.2	35.00	5024
III	13%ZrO_2_ + 87%(ZrB_2_–SiC)	303.1	0.174	8.289	820.0	49.43	5031
IV	3%ZrO_2_ + 97%(ZrB_2_–SiC)	284.4	0.155	7.913	824.0	54.78	5033
V	ZrB_2_–SiC	276.8	0.129	7.800	825.1	50.69	4716

## Data Availability

Not applicable.
